# Case Report: 3 cases of abdominal lesions with lower limb lymphedema—rare mutations in complex lymphatic anomalies

**DOI:** 10.3389/fonc.2025.1635659

**Published:** 2025-09-29

**Authors:** Yujia Zhang, Yaxin Jiang, Tong Qiu, Congxia Yang, Min Yang, Siyuan Chen, Yi Ji

**Affiliations:** ^1^ Department of Pediatric Surgery, West China Hospital, Sichuan University, Chengdu, China; ^2^ Department of Out-patient, West China Hospital, Sichuan University, Chengdu, Sichuan, China; ^3^ Med-X Center for Informatics, Sichuan University, Chengdu, China; ^4^ Department of Critical Care Medicine, West China Hospital, Sichuan University, Chengdu, China

**Keywords:** complex lymphatic anomalies, generalized lymphatic anomaly, kaposiform lymphangiomatosis, ALK, PDGFRA

## Abstract

Complex lymphatic anomalies (CLAs) are rare vascular anomalies characterized by abnormal proliferation of lymphatic and blood vessels, often resulting in multisystem involvement with heterogeneous clinical manifestations. We present 3 patients with CLAs exhibiting overlapping features of lower limb lymphedema and intra-abdominal vascular malformations, a phenotypic combination infrequently described in literature. Circulating tumor DNA (ctDNA) analysis in 2 cases detected somatic mutations at significantly higher variant allele frequencies compared to population databases, and mutant genes that were previously rare in vascular diseases were reported. These noninvasive molecular findings demonstrate diagnostic utility in identifying novel genotype-phenotype associations, providing insights into disease mechanisms and informing therapeutic strategies for atypical CLAs presentations. Our observations highlight ctDNA as a promising tool for refining diagnosis and guiding personalized management in rare vascular anomalies.

## 1 Introduction

Complex lymphatic anomalies (CLAs) are rare vasculopathies resulting from abnormal lymphatic development. They often lead to lymphatic leakage, and coagulation abnormalities. The clinical manifestations of CLAs typically appear in childhood and worsen during adolescence, affecting various tissues, such as the skin, bones, mediastinum, pleura, pericardium, liver, spleen, and gastrointestinal tract (1–3). The conditions include subtypes such as Generalized Lymphatic Anomaly (GLA), Kaposiform Lymphangiomatosis (KLA), and Central Conduction Lymphatic Anomaly (CCLA) (2).

This report focuses on three patients with abdominal lymphatic malformations complicated by lower limb lymphedema, including the identification of several rarely reported genetic mutations. By exploring these unique clinical features and genetic findings, we aim to elucidate the pathogenesis of CLAs, improve its classification, and inform the development of targeted treatment strategies for these complex conditions.

## 2 Case presentations

### 2.1 Patient 1

**Figure 1:**
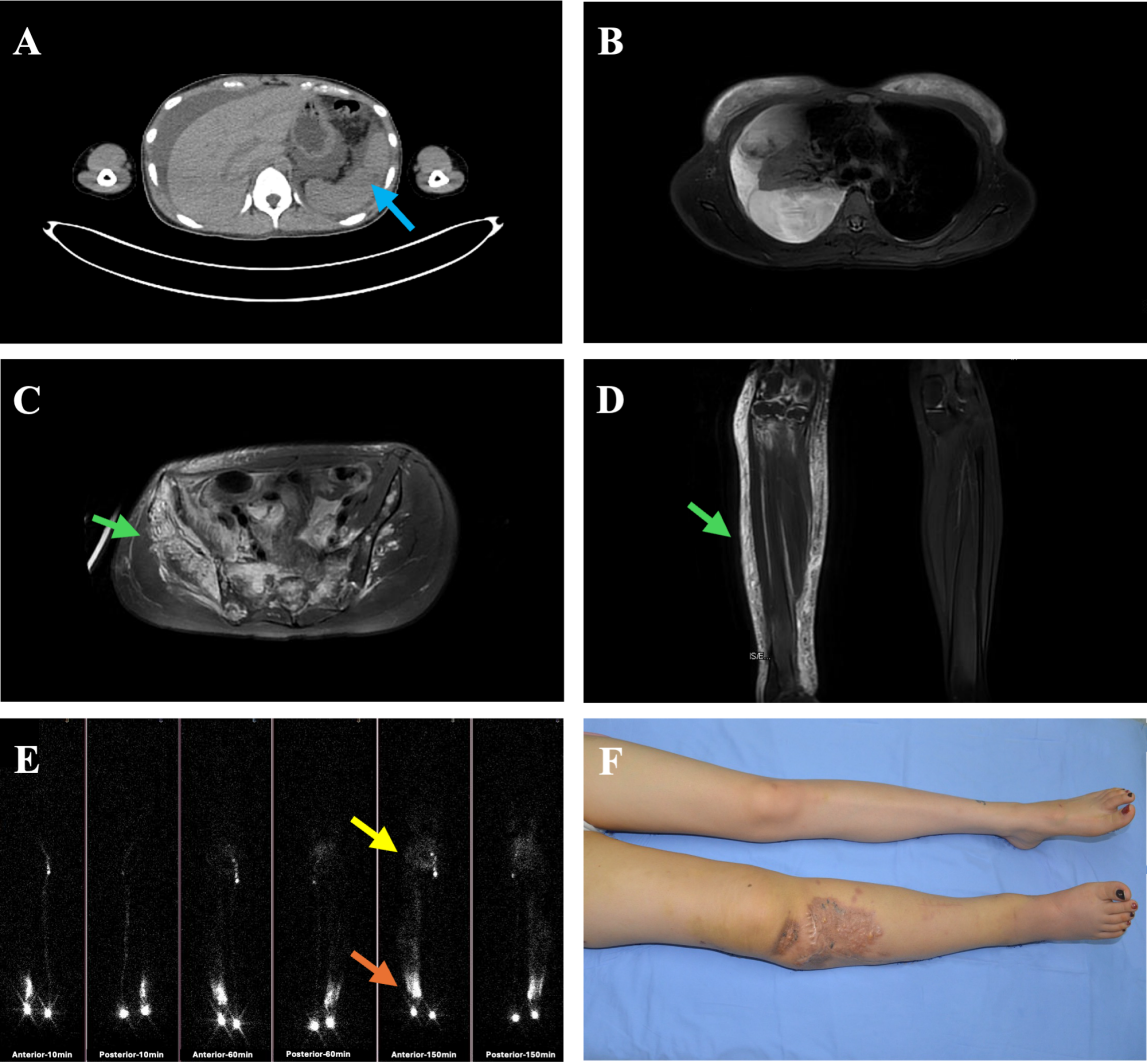
A female patient with Generalized Lymphatic Anomaly (GLA) manifested pleural and peritoneal effusions, cystic lesions of the spleen, and right lower extremity lymphedema. **(A)** Peritoneal effusion and nodular lesions in the splenic parenchyma are visualized on computed tomography (CT) (blue arrows). **(B)** Massive right-sided pleural effusion with significant compression of the right lung is evident on axial T2-weighted MRI. **(C, D)** High-signal-intensity lesions involving the dermis, subcutaneous tissue, and deep fascia of the right inguinal region and lower extremity are demonstrated on axial and coronal T2-weighted images, accompanied by perilesional soft tissue edema (green arrows). **(E)** Lymphoscintigraphy reveals non-visualization of right inguinal lymph nodes at 10, 60 and 150 minutes post-injection in the right foot (yellow arrows), along with abnormal tracer accumulation and cutaneous reflux in the right lower extremity (focal tracer retention highlighted by orange arrows). **(F)** Extensive right lower extremity swelling is documented in the clinical photograph.

A 23-year-old female patient first presented at age 3 with a dark mass on the anterior tibia of her right lower limb (**Figure 1**). The mass was surgically removed at a local hospital. One year postsurgery, she developed progressive edema in the right lower extremity with local mass recurrence and sporadic skin ecchymosis. Over the following 16 years, the edema spread throughout the entire right lower limb. To manage swelling, she used elastic compression socks. At 20 years old, she began experiencing lower back pain, and magnetic resonance imaging (MRI) revealed extensive bone destruction in the lumbar and thoracic vertebrae, pelvis, and ilium. Additionally, imaging revealed cystic lesions in the spleen, which were suspected to be lymphatic malformations.

At 22 years old, the patient suffered a severe lung infection, which worsened the edema in her right lower limb and led to repeated severe serous effusions, resulting in chest and abdominal infections, sepsis, and multiple organ failure. Although symptomatic anti-infective and supportive treatments temporarily improved her condition, she later developed recurrent aseptic inflammation in the chest and abdominal cavity. Based on the clinical manifestations and imaging examinations of Patient 1, we considered diagnosing this patient with GLA. Extensive bone destruction in the lumbar/thoracic vertebrae, pelvis, and ilium, alongside serous effusions, chest/abdominal infections, sepsis, and multiple organ failure collectively supported the diagnosis of GLA. Given the possible slow wound healing and ulceration caused by skin biopsy, we used circulating tumor DNA (ctDNA) sequencing to analyze peripheral blood samples from this patient for potential genetic mutations. NM_004304 (ALK) somatic mutation was detected in this patient: c.1918G> a (p.G640R), and the mutation abundance was 51.21%. Given the mutation abundance of over 50%, this gene mutation seems to be a germline mutation. However, the detection pattern of circulating tumor DNA (ctDNA) is designed to identify DNA fragments that are released into the bloodstream through apoptosis, necrosis, or active secretion by lesion cells. The mutations carried by these fragments typically reflect the genomic characteristics of the affected cells and are usually of somatic origin. No patients with similar symptoms or gene mutations were found in the family of patient 1.

Due to the lack of clear targeted drugs to treat vascular diseases associated with ALK gene mutations, we chose the more widely used sirolimus for treatment. The starting dose of oral sirolimus was 0.8 mg/m^2^ administered twice daily. Subsequently, oral sirolimus was titrated to achieve trough levels of 10–15 ng/ml. Patient 1 was referred to our center and began taking sirolimus orally and experienced significant improvement in symptoms during the first 6 months of treatment, after which the drug appeared to be less effective. Although the combination of oral diuretics (furosemide and spironolactone) and elastic compression stockings led to slight improvement in lower limb edema, the patient’s overall response was poor. She continued to experience recurring fluid accumulation and inflammation in the serosal space. Notably, her D-dimer level continued to rise despite the absence of significant coagulation dysfunction. No one else in her family has experienced similar symptoms.

### 2.2 Patient 2

**Figure 2:**
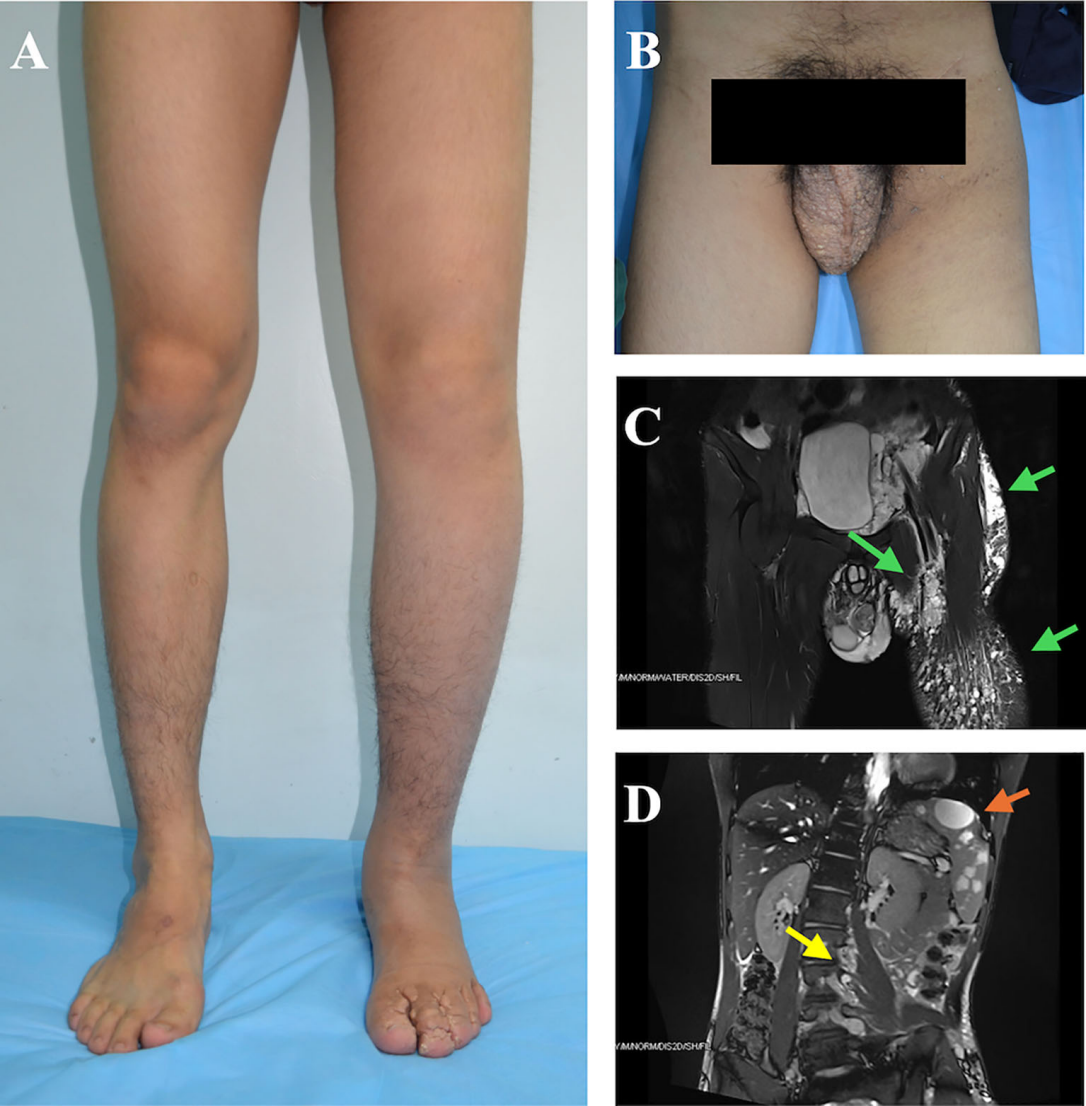
A male patient with generalized lymphatic anomaly (GLA) manifested cystic lesions of the spleen and lymphedema involving the left lower extremity and perineal region. **(A, B)** Clinical photographs demonstrate extensive swelling of the left lower extremity and perineal region, with prominent lymphorrhagia involving the penis, scrotum, and interdigital spaces of the left foot. Localized skin thickening and textural changes are evident. **(C)** Coronal T2-weighted MRI reveals multicystic and nodular high-signal-intensity lesions in the left perineal region and left lower extremity, involving the dermis, subcutaneous tissue, and deep fascia. Perilesional soft tissue edema is noted (green arrows). **(D)** Coronal T2-weighted MRI demonstrates multiple well-circumscribed, round hyperintense lesions within the spleen (orange arrows). Additionally, multiple tortuous dilated vascular structures are observed in the thoracolumbar spinal canal (yellow arrows).

An 18-year-old male patient was born with mild swelling of the perineum and left lower limb, which gradually worsened as he grew (**Figure 2**). At age 5, the patient was diagnosed with primary lymphedema of the left lower limb and perineum through lymphangiography. The swelling in his lower limbs was initially reduced with lymphatic venous anastomosis. However, at the age of 7, the swelling recurred and was managed with compression via elastic warmers and bandages.

Over the past year, the patient has experienced intermittent low back pain. Further examination revealed flexion and dilation of the thoracolumbar vascular vessels, as well as multiple lymphatic malformations in the spleen. ctDNA sequencing analysis of the peripheral blood of this patient did not reveal obvious gene mutations, which might be due to the limitation of sequencing depth. He was referred to our center for comprehensive evaluation and multidisciplinary treatment involving pediatric surgery, orthopedics, and other specialties. Based on Patient 2’s clinical manifestations, imaging findings, and other relevant evidence, we deemed the diagnosis of GLA appropriate. Widespread lymphatic malformations affecting multiple body sites, including the lower extremities and spleen, collectively support this diagnosis of GLA. The initial oral dose of sirolimus was set at 0.8 mg/m², administered twice daily. Subsequently, the dosage was adjusted to achieve a target trough level of 10–15 ng/ml. Oral sirolimus was initiated, leading to significant symptom improvement, particularly in reducing cutaneous lymphatic leakage and decreasing lower extremity swelling compared to before. Additionally, the neurosurgical team successfully treated several vascular malformations in the spinal canal through interventional embolization.

### 2.3 Patient 3

**Figure 3:**
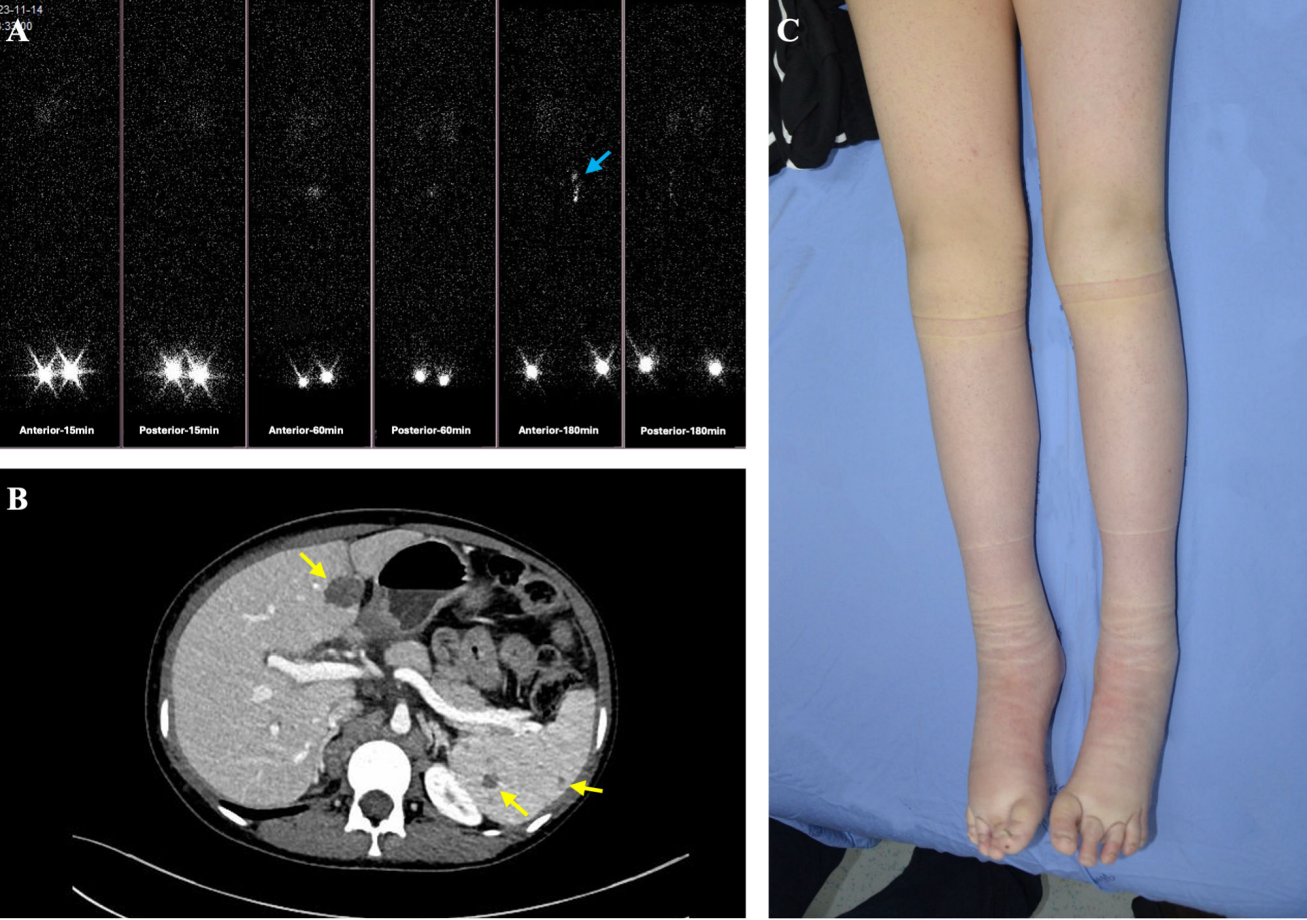
A female patient with generalized lymphatic anomaly (GLA) manifested multifocal cystic lesions in the liver and spleen, accompanied by bilateral lower extremity lymphedema. **(A)** Lymphoscintigraphy shows non-visualization of the right inguinal lymph nodes and delayed, faint visualization of the left inguinal lymph nodes at 15, 60, and 180 minutes post-injection in both feet (blue arrows). **(B)** Abdominal CT demonstrates multiple well-circumscribed, round hypodense lesions in the liver and spleen (yellow arrows). **(C)** Clinical photograph reveals extensive bilateral lower extremity swelling, more pronounced on the right, with localized skin thickening and textural changes.

A 13-year-old female presented with bilateral lower limb edema and persistent steatorrhea at birth (**Figure 3**). At age 3, she was diagnosed with primary lymphedema (PLE) and intestinal lymphangiectasia. Her lymphedema was managed with compression therapy using elastic bandages, while a medium-chain triglyceride (MCT) diet effectively resolved her steatorrhea. At age 11, she began experiencing intermittent right upper abdominal pain, which gradually increased in frequency and duration. No one else in her family has experienced similar symptoms.

Upon referral to our medical center, MRI identified multiple lymphatic malformations in the liver and spleen. Lymphoscintigraphy further revealed lymphatic drainage obstruction in the right lower limb and significant slowing in the left lower limb. Clinically, the presence of bilateral lower limb edema since birth, along with persistent steatorrhea aligns with the complex and multisystem nature of GLA. Imaging studies further corroborated this diagnosis: MRI revealed multiple lymphatic malformations in the liver and spleen, while lymphoscintigraphy demonstrated lymphatic drainage obstruction in the right lower limb and significant slowing in the left lower limb. These combined clinical and radiological features are characteristic of GLA, confirming the diagnosis. We used ctDNA sequencing to analyze peripheral blood samples from this patient for potential genetic mutations. ctDNA sequencing analysis of the peripheral blood of patient 3 revealed a somatic mutation of NM_006206 (PDGFRA): c.1415T>G (p.I472S), with a mutation abundance of 49.56%. A lymphedema specialist was consulted, and physical therapy techniques were introduced. However, despite these interventions, her PLE and abdominal pain showed only slight improvement. Similar symptoms or gene mutations were not identified in the family of Patient 3.

## 3 Discussion

CLAs are a group of rare disorders resulting from abnormal lymphatic system development and function, presenting significant diagnostic and therapeutic challenges. This study examines three patients with CLAs with uncommon manifestations, including abdominal lymphatic malformations accompanied by lower limb lymphedema—clinical features rarely reported in the literature. Genetic analysis further identified rare mutations in 2 patients, which are atypical in CLAs.

Although the etiology of CLAs remains poorly understood, recent research has begun to uncover its molecular basis. Mutations in genes such as *PIK3CA, NRAS*, and *KRAS* are frequently implicated (4, 5), providing potential targets for future investigations and therapeutic strategies. These findings highlight the need for a deeper understanding of the genetic and molecular mechanisms underlying CLAs to improve patient management and treatment outcomes.

The *anaplastic lymphoma kinase* (*ALK*) gene encodes a transmembrane receptor tyrosine kinase of the insulin receptor superfamily, playing a crucial role in embryonic and nervous system development. *ALK* mutations are commonly linked to non-small cell lung cancer, anaplastic large-cell lymphoma, neuroblastoma, and certain mesenchymal and brain tumor (6–8). However, reports of *ALK* mutations in vascular diseases are exceptionally rare. To date, only a few cases of CLAs and GLAs with *EML4:ALK* fusion mutations have been documented (9–11).

In this study, ctDNA analysis of Patient 1 identified an *ALK* mutation with a high mutation abundance of 51.21%, suggesting a substantial presence of *ALK*-mutated cells in circulation, likely originating from affected tissues. It is currently unclear whether the ALK p.G640R mutation found in our patients is pathogenic and thus a driving factor for the disease state, as no specific basic scientific experiments have been conducted to verify this. However, studies have shown that the ALK-1 signaling regulated by bone morphogenetic protein 9 (BMP-9) can inhibit lymphatic vessel formation (12). Studies on ALK-1-deficient mice have shown that lymphatic vessels proliferate excessively in multiple organs. Conversely, abnormal ALK activation may disrupt downstream pathways such as RAS/MAPK and PI3K-AKT, leading to abnormal lymphatic vessel development (13), as observed in our patients.

The *platelet-derived growth factor receptor alpha* (*PDGFRA*) gene encodes another receptor tyrosine kinase essential for cellular proliferation, differentiation, survival, and migration. While *PDGFRA* mutations are well-documented in malignancies such as gastrointestinal stromal tumors (GISTs) and acute lymphoblastic leukemia, their role in vascular diseases remains largely unexplored (14, 15). Mutations in the kinase domain of *PDGFRA* can lead to dysregulated receptor activation, driving tumorigenesis through pathways such as *MAPK* and *PI3K/AKT (* 16, 17). In GISTs, *PDGFRA*-driven tumor progression has led to the development of targeted therapies, including tyrosine kinase inhibitors (TKIs) (18, 19). These therapies provide valuable insights into potential treatment strategies for patients with CLAs with *PDGFRA* mutations. While the mechanisms underlying *PDGFRA*-mediated lymphatic anomalies remain poorly understood, investigating *PDGFRA*-targeted therapies for CLAs could offer new therapeutic options, particularly for patients with overlapping genetic or pathological features.

The diagnosis of CLAs requires a multidisciplinary approach that integrates clinical evaluation, imaging, tissue biopsy, and genetic testing (1, 3). MRI plays a central role in assessing lymphatic malformations, offering detailed visualization of their size, distribution, and relationship to surrounding structures (3). Additionally, functional imaging techniques such as lymphography and lymphoscintigraphy are essential for evaluating lymphatic drainage, detecting obstructions, and identifying areas of stasis or leakage (20). Tissue biopsies and genetic testing are crucial for distinguishing CLAs from other vascular abnormalities and related conditions. Beyond aiding in diagnosis, genetic testing facilitates personalized treatment by identifying specific mutations that may guide targeted therapies. ctDNA analysis detects DNA fragments that are apoptotic, necrotic or actively secreted and released into the bloodstream by diseased cells. It provides a noninvasive alternative to traditional biopsies by detecting genetic alterations from small DNA fragments shed into the bloodstream (21, 22). This method minimizes patient trauma, particularly for individuals with lymphedema, where wound healing may be impaired.

In this study, peripheral blood ctDNA testing was utilized to identify genetic mutations in patients with CLAs, circumventing the risks associated with intraperitoneal lesion biopsy. Mutations were detected in nearly half of the ctDNA samples from Patients 1 and 3, a higher detection rate than previously reported for vascular diseases using similar techniques (23, 24). These findings suggest that ctDNA analysis could serve as a valuable diagnostic tool for vascular anomalies.

However, the lower mutation rate in the blood of patients with vascular anomalies compared to those with solid tumors presents challenges (23). Current sequencing panels and depths are primarily designed for oncology applications and may not be optimized for detecting vascular disease-related mutations, increasing the risk of false negatives. This underscores the need for further research to refine sequencing technologies for vascular conditions. Developing targeted panels and adjusting sequencing depth could enhance the sensitivity and specificity of ctDNA testing, providing a viable diagnostic alternative for patients in whom tissue biopsy is not feasible. Continued investigation in this area is essential to improve diagnostic accuracy and expand clinical applications for CLAs.

Despite increasing recognition of these rare diseases, the treatment of CLAs remains challenging. Management typically requires an individualized approach, often involving multidisciplinary collaboration to address each patient’s specific condition. Current treatment strategies include both pharmacological and surgical interventions aimed at symptom control and complication management. Drug therapies primarily target lymphangiogenesis and disease progression. Recent studies have demonstrated the efficacy of targeted treatments such as sirolimus, trametinib, and alpelisib in symptom control and improving quality of life (25–28). These agents function by modulating key signaling pathways to inhibit the proliferation of abnormal lymphatic endothelial cells, offering promising therapeutic options for patients with CLAs (29).

In this study, Patient 1 initially responded well to oral sirolimus, experiencing a reduction in chest and abdominal fluid accumulation and improved lower limb edema with concurrent physical compression therapy. However, after six months of continuous treatment, the drug’s effectiveness declined, leading to recurrent aseptic inflammation in the abdominal cavity, dyspnea, joint pain, and other symptoms. Patient 2 showed mild improvement in lower limb edema following sirolimus treatment, with no further cutaneous lymphatic leakage. However, abdominal lesions persisted, necessitating additional intervention. The patient underwent interventional embolization surgery to address spinal vascular malformations. Patient 3’s management relied on physical therapy to control lower limb lymphedema and an MCT diet to prevent steatosis. Unlike the other two cases, pharmacological intervention was not utilized.

Although numerous studies have demonstrated the efficacy of sirolimus in treating (26, 28, 30, 31) CLAs, the therapeutic response observed in this study did not always meet expectations. This underscores the need for a more comprehensive understanding of disease pathogenesis and treatment strategies. Additionally, the gene mutations identified in the peripheral blood of Patient 1 and Patient 3 were largely novel, with minimal prior documentation in the literature. Their mutation frequencies also differed significantly from those reported in other studies (23, 32), and their pathogenic mechanisms remain unclear due to the lack of experimental validation. Consequently, it is uncertain whether these mutations directly contribute to the patients’ clinical manifestations. Given this uncertainty, targeted therapies related to ALK or PDGFRA mutations were not utilized in this study, highlighting the necessity of further research into precision treatment for patients with CLAs with rare mutations.

In addition, the decreased drug response of Patient 1 to sirolimus has also drawn attention. We repeatedly tested the patient’s target trough levels and adjusted the drug dosage based on the results. Despite maintaining a consistent target trough levels, the patient’s response to the drug was still not obvious. Therefore, we speculate that it may be related to sirolimus resistance. Previous studies have also found similar phenomena in patients with kaposiform hemangioendothelioma. This resistance may be associated with the complex negative feedback loops between mTORC1 and its upstream signaling pathways (33), warranting further investigation into combination therapies or alternative targeted approaches.

## 4 Conclusion

This study presents three cases of CLAs associated with lower limb lymphedema, with two patients exhibiting rare genetic mutations. The relationship between these mutations and the onset and progression of CLAs remains unclear. The relatively high frequency of these mutations observed in this study is unexpected and warrants further investigation. Additional case reports and larger patient cohorts are needed to validate these findings and explore their clinical significance. Given the rarity and complexity of CLAs, our understanding of its pathogenesis, disease progression, and optimal treatment strategies remains limited. Future research should focus on elucidating the molecular mechanisms underlying CLAs, improving diagnostic accuracy through advanced genetic testing, and developing targeted therapies tailored to patients with specific mutations. In addition, ctDNA appears to have demonstrated its role in the diagnosis of vascular diseases, and it is hoped that this non-invasive test will help identify pathogenic variants and guide medical management to improve the accuracy of treatment in these complex medical conditions.

## Data Availability

The raw data supporting the conclusions of this article will be made available by the authors, without undue reservation.
